# Supplementary material for: First trimester “clean catch” urine and vaginal swab sample distinct microbiological niches
Source: Microbiol Spectr. 2023 Dec 13;12(1):e02638-23. doi: 10.1128/spectrum.02638-23 (PMC10782990; doi:10.1128/spectrum.02638-23)
Supplement: Table S1 — Culture protocols: SUC versus EQUC. [file spectrum.02638-23-s0001.docx]

**Supplemental Table 1: Culture Protocols SUC versus EQUC**

| **Protocol** | **Volume**  **Plated** | **Medium Composition** | **Atmospheric**  **Environment** | **Incubation**  **Time** |
| --- | --- | --- | --- | --- |
| Standard  Urine Culture | 1 µL | Blood agar  MacConkey agar | Aerobic | 24 hours  35 degrees |
| Expanded Quantitative  Urine Culture | 100 µL | Blood agar  Chocolate agar  Aerobic Blood agar  CNA agar  MacConkey agar | Aerobic  CO_2_  Anaerobic | 48 hours  35 degrees |
